# Is OperaVOX a clinically useful tool for the assessment of voice in a general ENT clinic?

**DOI:** 10.1186/s12901-017-0037-9

**Published:** 2017-04-21

**Authors:** Richard Teck Kee Siau, Jay Goswamy, Sue Jones, Sadie Khwaja

**Affiliations:** 1 Department of Otolaryngology – Head and Neck Surgery, University Hospital of South Manchester NHS Foundation Trust, Wythenshawe Hospital, Manchester, UK; 2 Department of Speech and Language Therapy, University Hospital of South Manchester NHS Foundation Trust, Manchester, UK

**Keywords:** OperaVOX, Dysphonia, Voice assessment, Portable voice analysis, Perceptual voice analysis, Acoustic voice analysis, GRBAS

## Abstract

**Background:**

Objective acoustic analysis is a key component of multidimensional voice assessment. OperaVOX is an iOS app which has been shown to be comparable to Multi Dimensional Voice Program for most principal measures of vocal function. As a relatively cheap, portable and easily accessible form of acoustic analysis, OperaVOX may be more clinically useful than laboratory-based software in many situations. This study aims to determine whether correlation exists between acoustic measurements obtained using OperaVOX, and perceptual evaluation of voice.

**Methods:**

Forty-four voices from the multidisciplinary voice clinic were examined. Each voice was assessed blindly by a single experienced voice therapist using the GRBAS scale, and analysed using OperaVOX. The Spearman rank correlation co-efficient was calculated between each element of the GRBAS scale and acoustic measurements obtained by OperaVOX.

**Results:**

Significant correlations were identified between GRBAS scores and OperaVOX parameters. Grade correlated significantly with jitter (*ρ* = 0.495, *p* < 0.05), shimmer (*ρ* = 0.385, *p* < 0.05), noise-to-harmonic ratio (NHR; *ρ* = 0.526, *p* < 0.05) and maximum phonation time (MPT; *ρ* = −0.415, *p* < 0.05). Roughness did not correlate with any of the measured variables. Breathiness correlated significantly with jitter (*ρ* = 0.342, *p* < 0.05), NHR (*ρ* = 0.344, *p* < 0.05) and MPT (*ρ* = −0.336, *p* < 0.05). Aesthenia correlated with NHR (*ρ* = 0.413, *p* < 0.05) and MPT (*ρ* = −0.399, *p* < 0.05). Strain correlated with Jitter (*ρ* = 0.560, *p* < 0.05), NHR (*ρ* = 0.600, *p* < 0.05) and MPT (*ρ* = −0.356, *p* < 0.05).

**Conclusions:**

OperaVOX provides objective acoustic analysis which has shown statistically significant correlation to perceptual evaluation using the GRBAS scale. The accessibility of the software package makes it possible for a wide range of health practitioners, e.g. general ENT surgeons, vascular surgeons, thyroid surgeons and cardiothoracic surgeons to objectively monitor outcomes and complications of surgical procedures that may affect vocal function. Given the increasing requirement for surgeons to monitor their outcomes as part of the move towards ‘surgeon reported outcomes’ this may become an invaluable tool towards that goal.

## Background

There is an increasing need for quantified measures of vocal function; this is required for the patient, the clinician and local voice units to measure outcomes following treatments for a full spectrum of voice disorders. Furthermore, a standardized protocol for assessment of voice is required in order to assess and compare voice treatments and is thus integral to research in the field of laryngology. There is general agreement that assessment of voice should be multidimensional – both perceptual measures and acoustic analyses must be considered, in addition to videostroboscopy and subjective rating by the patient [1].

Despite extensive research in the domain, there is currently no single widely accepted standardized technique of objective voice evaluation. The Multi Dimensional Voice Programme acoustic analysis system (MDVP, KayPentax, USA) is a voice analysis software package widely used in voice clinics and in published voice research. OperaVOX (On PErson RApid VOice eXaminer, Oxford Research Wave Ltd, UK) is a portable voice analysis software package designed for use with iOS devices such as iPod touch, iPhone and iPad (Apple, USA). OperaVOX has been shown to be reliable and comparable to MDVP for most principal measures of vocal function, with the exception of noise-to-harmonics ratio [2].

Despite the emergence of more technical and objective evaluations of voice using software such as OperaVOX, perceptual evaluation of voice remains an essential tool for the assessment of voice quality in the clinical setting. The GRBAS scale developed by the Japan Society of Logopaedics and Phoniatrics is a four-point ordinal scale containing five well-defined parameters: Grade, Roughness, Breathiness, Aesthenia and Strain. Although there is no internationally accepted perceptual evaluation protocol, the GRBAS scale is validated with acceptable intra-observer and inter-observer variance, and is the most widely used perceptual rating system [3].

A small number of studies in the current literature investigate the relationship between perceptual evaluation of voice quality and acoustic measurements. However, all of these studies use hospital-based computer software for acoustic analysis. These software packages are typically only accessible to multidisciplinary voice clinics in the UK. As a relatively cheap, portable and easily accessible form of acoustic analysis, OperaVOX may be more clinically useful than laboratory-based software in many situations. To our knowledge, this is the first study to analyse the correlation between acoustic measurements taken by OperaVOX and perceptual evaluation of dysphonia.

## Methods

### Participant selection and assessment

Study participants were recruited by convenience sampling from patients presenting to the multidisciplinary voice clinic at University Hospital of South Manchester between August 2014 and January 2015. Only patients who were offered phonosurgery were included. All selected patients consented verbally to be included in the study and vocal tasks were carried out in an outpatient clinic room, with background noise level monitoring.

### Study software

OperaVOX is an iOS application that facilitates portable acoustic analysis of voice samples. At the time of the present study, there are three versions of OperaVOX available: OperaVOX Lite (free for personal use), OperaVOX Personal (USD $42.99, GBP £32.99) and OperaVOX Multi (USD $399.99, GBP £299.99). All three versions of the software record and analyse vocal samples identically. In this study, the OperaVOX Personal software was installed on a second-generation iPad mini with Retina Display (Apple, Cupertino, USA). Another iOS app, Decibel 10th (SkyPaw Co. Ltd, Vietnam) was used to measure the background sound pressure level, which ranged from 39 to 55 dBSPL.

Participants were prompted by OperaVOX to vocalize the sustained vowel/a/for five seconds. This was performed three times to obtain measures of jitter, shimmer and noise-to-harmonics ratio (NHR). Following this, the patient was again prompted to take a normal inspiration and vocalize the vowel/a/for as long as possible. This task was repeated to obtain the best reading from three attempts: the maximum phonation time (MPT).

### Perceptual evaluation

Participants were then asked to read the “Rainbow Passage”, a commonly used phonetically balanced text. Digital 16 bit, 44.1 kHz uncompressed wave format recordings of this passage were anonymised and scored using the five parameters of the GRBAS scale by a single experienced voice therapist: Grade = overall perceived degree of dysphonia, Roughness = irregular fluctuation of the fundamental frequency, Breathiness = turbulence due to leakage of air, Asthenia = weakness of voice, and Strain = perceived excess effort. Each parameter was scored using an ordinal scale of 0 to 3: 0 = normal, 1 = slight disturbance, 2 = moderate disturbance, and 3 = severe disturbance.

### Statistical analysis

Acoustic parameters measured using OperaVOX were compared with each element of the GRBAS perceptual evaluation using the Spearman rank correlation co-efficient, ρ. Spearman’s ρ values range from -1 to +1, representing perfect negative and positive monotonic correlations respectively. Statistical analysis was performed using SPSS (IBM Corporation, New York, USA) installed on a MacBook Air (Apple, Cupertino, USA) running Mac OS X 10.9.1.

## Results

Forty-four voice samples were obtained from 29 patients with voice disorders undergoing surgical treatment, with 15 repeat samples recorded at the first post-operative follow-up clinic. Post-operative voice assessments were performed between 14 and 147 days post-operatively (mean 39.5 days). 21 samples were obtained from male patients and 23 from female patients. Mean age was 60.1 years (SD 17.3). Diagnoses are listed below in Table 1.

**Table 1:** Diagnoses of voices examined

Diagnosis	n
Vocal fold palsy	22
Vocal fold lesions	14
Spasmodic dysphonia	5
Reinke's oedema	3

**Fig. 1:**
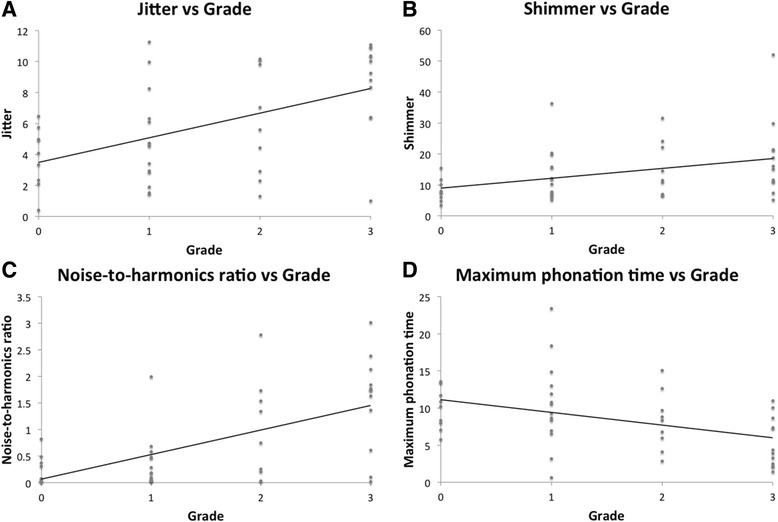
Correlation between OperaVOX acoustic measurements and overall grade of voice. **a**: Correlation between jitter and grade. **b**: Correlation between shimmer and grade. **c**: Correlation between noise-to-harmonics ratio and grade. **d**: Correlation between maximum phonation time and grade

Significant correlations were identified between OperaVOX parameters and GRBAS (Table 2). Grade correlated significantly with jitter, shimmer, NHR and MPT (Fig. 1). Roughness did not correlate with any of the measured variables. Breathiness correlated significantly with jitter, NHR and MPT but not shimmer. Asthenia correlated with jitter, NHR and MPT but not shimmer. Strain correlated with all four measured OperaVOX variables.

**Table 2:** Spearman rank correlation co-efficients between OperaVOX measurements and GRBAS parameters

	Jitter	Shimmer	NHR	MPT
Grade	0.495* *p* = 0.001	0.385* *p* = 0.01	0.526* *p* = 0	−0.415* *p* = 0.005
Roughness	0.199 *p* = 0.196	0.239 *p* = 0.118	0.272 0.074	−0.074 *p* = 0.635
Breathiness	0.342* *p* = 0.023	0.275 *p* = 0.071	0.344* *p* = 0.022	−0.336* *p* = 0.026
Aesthenia	0.445* *p* = 0.002	0.220 *p* = 0.152	0.413* *p* = 0.005	−0.399* *p* = 0.007
Strain	0.560* *p* = 0	0.411* *p* = 0.006	0.600* *p* = 0	−0.356* *p* = 0.018

Statistically significant correlations are highlighted with an asterisk. Correlation co-efficient size interpretation: 0.0–0.3, negligible correlation; 0.3–0.5, low correlation; 0.5–0.7, moderate correlation; 0.7–0.9, high correlation, 0.9–1.0: very high correlation [18]. *NHR* noise-to-harmonic ratio, *MPT* maximum phonation time

## Discussion

These results describe the relationship between objective acoustic measurements taken using OperaVOX and perceptual evaluation of voice quality using the GRBAS scale. Previous studies have examined the relationship between objective and perceptual measures of voice quality, but have used laboratory-based equipment and software, inaccessible to most clinicians and patients. This study is the first to examine this correlation using OperaVOX, the unique benefits of which are ergonomics, portability and accessibility.

Jitter, shimmer and NHR are measures of vocal perturbation and harmonicity, and thus if measured reliably, may be predictors of severity of dysphonia. Many studies have investigated the relationship between these three parameters and perceived dysphonia. Ma et al. found that jitter and shimmer correlate well with perceived overall dysphonia [4]. De Krom showed that NHR is a strong predictor of listener-rated roughness and breathiness [5]. Furthermore Martin et al. found that NHR significantly correlates with dysphonic severity of rough voices, reporting a correlation co-efficient (R^2^) of 0.77 between NHR and roughness [6]. Dejonkere et al found that overall grade of voice correlated with both shimmer and NHR and that roughness correlated significantly with jitter, and breathiness with shimmer [7]. Bhuta et al found significant correlations between overall grade and NHR and between roughness and NHR. However acoustic parameters of jitter and shimmer did not significantly correlate with GRBAS [8]. Furthermore Wolfe et al. found no strong correlations between any of these perturbation measures with perceptually-rated dysphonic severity [9]. Compared with these studies, we found significant low-to-moderate correlation between all three perturbation and harmonicity parameters measured by OperaVOX and overall grade of dysphonia, as well as various components of the GRBAS scale.

Maximum phonation time is a widely used aerodynamic measure of laryngeal efficiency and vocal stability. Although it may be confounded by vital capacity, it has long been used by speech pathologists as a simple method of recording the acoustic performance of pathological voices. MPT has a high level of reliability and has been used alone to quantify severity of dysphonia and to measure outcomes of voice therapies [10, 11]. Yu et al found that MPT correlated significantly with overall grade of dysphonia [12]. Our findings support this, and in addition we report that MPT measured using OperaVOX shows low correlation with ratings of breathiness, asthenia and strain.

Despite the multitude of studies examining the relationship between acoustic parameters and perceptual assessment, the relationship remains unclear and there lacks robust agreement between studies. Several authors have consequently urged caution over the increased reliance on objective measures of dysphonia [9, 13]. We propose that the lack of agreement between studies, including results of our present study, can be partially accounted for by the variability in study protocols, particularly the different software packages used and method of perceptual evaluation examined. We have chosen OperaVOX, an iOS programme that can be installed on any iPad, iPhone or iPod mini device to obtain acoustic analysis, and the GRBAS scale as it is widely considered the gold standard tool for perceptual evaluation, with low intra-rater and inter-rater variability [3]. The acoustic analysis obtained using OperaVOX has previously been shown to be reliable, with data comparable to that obtained using MDVP, a popular laboratory software package used in voice clinics worldwide [2].

Wuyts et al. have derived the Dysphonia Severity Index (DSI), a multiparametric measure for the severity of dysphonia, which combines four objective measures: fundamental frequency, lowest intensity, MPT and jitter.[14] The DSI has been constructed so that scores correlate with overall Grade of dysphonia as rated by a jury of expert raters using the GRBAS scale. The DSI has been shown to have good interobserver and test-retest variability, and has also been shown to be a measure of severity of dysphonia [15, 16]. OperaVOX measures three of the four objective parameters used for the DSI, but does not include the lowest intensity. We suggest that in future iterations of OperaVOX, this additional parameter be added and the DSI calculation could be performed as part of the automatic voice analysis to provide a useful objective measurement of overall severity of dysphonia which would be easily interpreted by healthcare providers and patients alike.

### Limitations

In our study, a single rater was used for perceptual evaluation of vocal quality. In clinical practice, often only a single rater grades the voice subjectively using this tool; we therefore felt it was reasonable to use only a single rater for this. However additional raters would allow for interrater reliability analysis and further studies should use this to increase validity of results.

The mouth-to-microphone distance used for vocal analysis and recording was not specified – instead the participants were asked to hold out the iPad at arm’s length. A lanyard of specified length, for example 50 cm, could be worn around the participant’s neck to standardize this. Use of a lanyard is not suggested in the OperaVOX instructions, and thus patients would be unlikely to use such standardization on their own in day-to-day use.

The study only included patients selected to undergo phonosurgery. Vocal assessments were performed twice on fifteen patients: before and after surgery. We do not feel this affects the validity of our findings as the main purpose was to assess the relationship between subjective and objective assessments of voice, and the inclusion of repeat samples increases the sample size of our study. In retrospect however, patients not undergoing surgery could have been included in the study to increase sample size and to include a wider variety of voice disorders.

## Conclusions

We have shown correlations between many of the acoustic measurements and the elements of the GRBAS evaluation. The strongest correlations identified are between grade and NHR (*ρ* = 0.526) and between strain and jitter (*ρ* = 0.560). Significantly, all four acoustic parameters examined correlate with overall grade of dysphonia. Given its wide availability and ease of use, we suggest that OperaVOX may be used widely in the voice clinic by Speech pathologists, Laryngologists, General Otolaryngologists and patients alike as part of a multi-dimensional assessment of vocal function to assess the effect of therapies. The accessibility of the software package makes it possible for other health practitioners, e.g. general ENT surgeons, vascular surgeons, thyroid surgeons and cardiothoracic surgeons to objectively monitor outcomes and complications of a wide range of surgical procedures that potentially affect vocal function. Given the increasing requirement for surgeons to monitor their outcomes as part of the move towards ‘surgeon reported outcomes’ this may become an invaluable tool towards that goal. Although our work has increased the clinical validity of OperaVOX analysis, this software remains relatively new and more research needs to be done to confirm its utility.

## Abbreviations

MDVP: Multi dimensional voice programme
NHR: Noise-to-harmonics ratio
MPT: Maximum phonation time
DSI: Dysphonia severity index
